# Study on the influencing factors of forest-based health and wellness services from the supply and demand perspective

**DOI:** 10.3389/fpubh.2025.1665320

**Published:** 2025-09-19

**Authors:** Kunjing Wei, Geyao Zhou, Xianfeng Zhou, Yufang Zhang, Qiuyue Liao

**Affiliations:** ^1^School of Medicine and Health Management, Guizhou Medical University, Anshun, Guizhou, China; ^2^One Health Institute of Guizhou Medical University, Anshun, Guizhou, China; ^3^School of Public Health, Guizhou Medical University, Anshun, Guizhou, China

**Keywords:** supply and demand side perspective, forest-based health and wellness, service, influencing factors, Guizhou Province

## Abstract

**Objective:**

This study investigates the key factors influencing the development of forest-based health and wellness (FHW) services in Guizhou Province, China, from both supply- and demand-side perspectives.

**Methods:**

A purposive sample of 242 staff members and 420 consumers from 17 FHW bases was surveyed between March and June 2024. Data were analyzed using binary logistic regression for supply-side factors and ordered logistic regression for demand-side determinants, following reliability and validity testing of the questionnaires.

**Findings:**

On the supply side, the effectiveness of health management programs, adequacy of health facilities, and implementation of health education activities significantly affected service delivery. On the demand side, consumer satisfaction was shaped by instructor professionalism, perceived health benefits, the layout of leisure facilities, staff courtesy, and access to health product purchase channels.

**Conclusion:**

To advance the sustainable development of FHW, we propose a coordinated agenda across stakeholders. On the supply side, providers should strengthen the scientific rigor and implementation fidelity of health-management programs, upgrade and complete wellness infrastructure, and institutionalize routine health-education activities. On the demand side, priorities include enhancing the professional competence of wellness interpreters/guides and therapists; increasing consumers’ perceived health gains; optimizing the siting and accessibility of rest pavilions and related amenities; and improving courteous, patient-centered service as well as the availability and accessibility of health-product purchasing channels. For policymakers, stronger interdepartmental coordination is needed to support and enable the effective implementation of these measures.

**Novelty:**

This study provides one of the first comprehensive analyses combining supply and demand perspectives in FHW services, offering evidence-based recommendations for policy, practice, and industry development in emerging health economies.

## 1 Introduction

In recent years, chronic lifestyle-related diseases such as sub-health conditions, cardiovascular diseases, mental disorders, and malignant tumors, as well as population aging, have gradually become critical challenges affecting social development in various countries (1–5). Due to their unique therapeutic effects, forests have been considered an effective solution to these health challenges (6, 7), particularly with substantial potential in addressing the health demands of older adults and promoting the growth of the silver economy (8, 9). Studies have confirmed that forests play a crucial role in preventing non-communicable diseases, including hypertension and heart disease (10). Moreover, forest environments contribute positively to physiological health, mitigating numerous health issues associated with lifestyle factors, such as alleviating depressive symptoms, reducing anxiety, alleviating burnout syndrome, and relieving daily stress (11–17). In this context, people have started to pursue a healthy lifestyle that is in harmony with nature and focused on low-carbon and environmentally friendly practices, leading to the rise of FHW services (18).

Tracing back the development history of FHW, it originated in Germany (19), where individuals primarily engaged their senses, such as breathing fresh forest air, observing plant colors, and listening to birdsong to achieve calming effects and stress relief through inhalation of volatile aromatic substances in forests (20, 21). In Canada, Europe and America, et al. FHW is referred to as “nature experience” or “interaction with nature” (22). In Japan, it is known as “Shinrin-yoku” or “forest bathing,” in promoting forest bathing, Japan integrated it with medical science, subsequently introducing concepts such as “forest therapy” and “forest medicine” (23, 24). In 2012, China began introducing and promoting the Japanese concept of forest therapy. By 2013, the term “forest therapy” (translated as “森林康养” in Chinese) was officially adopted in China (25). Despite variations in terminology, the essence of FHW remains consistent—activities conducted within forest environments that benefit human physical and mental health (20).

In China, forest-based health and wellness (FHW) has emerged as a new, integrative sector at the nexus of forestry and health promotion, playing a pivotal role in advancing the Healthy China strategy and the “Two Mountains” principle that “lucid waters and lush mountains are invaluable assets” (26). Conceptually, FHW is a distinctive, comprehensive wellness system grounded in high-quality forest resources—such as forest landscapes, forest foods, and forest eco-culture—that integrates modern medicine with traditional Chinese medicine. Guided by the ethos of being people-centered, forest-grounded, wellness-oriented, and health-anchored, FHW deploys appropriate leisure, medical, and fitness facilities in forest settings to deliver activities that promote physical and mental well-being, enhance fitness, restore health, delay aging, prevent disease, and provide restorative recreation (27, 28). The resulting FHW services represent the practical embodiment of this system: relying on the forest ecological environment, integrates the above-mentioned resources and concepts, and provides the public with a comprehensive experience that promotes physical and mental health through a variety of activities (29). As a country rich in forest resources, China is comprehensively supporting the construction of the FHW industry through policy guidance and practice-led pilots, steadily advancing and optimizing sectoral development (30). By 2024, more than 3,000 FHW bases had been established nationwide, receiving over 400 million visits and laying an initial foundation for scale. In parallel, many regions are exploring “forest+” models that embed FHW within tourism, healthcare, and cultural industries to enriching service content and construction modalities.

Recent years have witnessed initial advances in research on FHW services. Existing studies have primarily focused on exploring the developmental potential of FHW services within nature reserves (31), analyzing key factors influencing satisfaction with FHW service experiences (32), assessing the relationship between service functionality and consumer demands (33), and identifying various consumer demand types for FHW services (34). Although prior studies have examined, to varying degrees, the service attributes of FHW and the needs of consumers, most adopt a single-dimensional lens; in-depth analyses that integrate both the supply- and demand-side determinants of FHW service development remain scarce. This omission risks leaving relevant agencies without a comprehensive and systematic evidence base for designing optimization strategies and evaluating health benefits, thereby constraining the scientific development and broader diffusion of the sector. Accordingly, this study undertakes a dual supply–demand analysis of the key factors shaping FHW services, a perspective with clear practical significance for improving service quality, meeting heterogeneous needs, and advancing the high-quality development of the industry. Guizhou Province, as a multi-ethnic region with abundant forest resources and medicinal plants, possesses unique ecological advantages conducive to developing a robust FHW industry (35), and has already established a relatively mature and comprehensive industrial system for FHW (36). Given this context, the present study takes FHW bases in Guizhou Province as examples, deeply analyzing the influencing factors from the perspectives of both staff members and consumers, and proposes targeted recommendations accordingly. This study aims to serve as a valuable reference for other countries and other regions possessing similar resources in developing FHW services.

## 2 Methods

### 2.1 Research participants

Based on the level of economic development and the construction status of FHW bases, this study selected Guiyang City, Zunyi City, Tongren City, Qiandongnan Prefecture, and Qianxinan Prefecture as the survey areas. Using purposive sampling, 17 FHW bases were selected, and an on-site questionnaire survey was conducted with both staff members and consumers between March and June 2024.

#### 2.1.1 Inclusion criteria for staff members were as follows

1. Affiliation with departments such as administrative management, logistical services, facility maintenance, publicity and marketing, et al.;
2. Awareness of and participation in FHW services or projects at their respective bases;
3. Informed consent and voluntary participation in this study.

#### 2.1.2 Inclusion criteria for consumers were as follows

1. Ability to independently complete the questionnaire;
2. Prior experience of FHW services at the selected bases;
3. Informed consent and voluntary participation in this study.

### 2.2 Research methods

During the questionnaire design phase, this study conducted a systematic review of relevant domestic and international literature and policy documents. Consultations with subject-matter experts were also carried out, and based on their feedback, the questionnaire was revised and a pilot survey was conducted, ultimately resulting in the finalized version. The content of the survey was as follows:

**Staff questionnaire includes**: basic information; understanding of the concept and functions of forest health care; awareness of relevant policies and regulations related to forest health care; knowledge of the operational model of the forest health care industry within the scenic area; evaluation of the attitudes of management staff toward the development of the forest health care industry; evaluation of attitudes of the residents toward the development of the forest health care industry; perceptions of the effectiveness of health management program implementation; and whether the staff perceives their work as meaningful, among other questions.

**Consumer questionnaire includes**: basic information; satisfaction with natural biodiversity resources; satisfaction with distinctive cultural resources; satisfaction with the comfort of the ecological environment; satisfaction with promotional materials and tourist maps; satisfaction with the designated forest wellness zones; satisfaction with wellness service facilities; satisfaction with the professionalism of wellness interpreters or therapists; perceived politeness and service quality of staff; perceived diversity of wellness programs; and the overall experiential quality of wellness activities.

The questionnaires were distributed and collected through face-to-face interactions with both staff members and consumers. In total, 242 staff questionnaires were recovered, with an effective response rate of 86.43%, and 420 consumer questionnaires were recovered, with an effective response rate of 93.33%. To ensure the overall validity and reliability of the questionnaires, this study conducted reliability and validity testing. The results showed that the Cronbach’s *α* coefficient was 0.846 for the staff questionnaire and 0.964 for the consumer questionnaire, indicating good internal consistency for both instruments. Exploratory factor analysis further revealed a Kaiser-Meyer-Olkin (KMO) value of 0.831 for the staff questionnaire and a Bartlett’s test of sphericity result of χ^2^ = 882.576 (*p* < 0.001); for the consumer questionnaire, the KMO value was 0.967 and Bartlett’s test result was χ^2^ = 6612.939 (*p* < 0.001). These results indicate strong structural relationships among the variables, confirming that both questionnaires possessed good construct validity.

### 2.3 Statistical methods

Statistical analyses were conducted with SPSS 22.0. Categorical variables were expressed as composition ratios and percentages. On the supply side, a χ^2^ test was used for univariate analysis to identify factors influencing the effective implementation of FHW services in Guizhou Province. Variables that showed statistical significance in the univariate analysis were included in a multivariate analysis using binary logistic regression. On the demand side, a χ^2^ test was also employed for univariate analysis to explore factors influencing overall consumer satisfaction. Variables with statistical significance were subsequently analyzed using ordered logistic regression. *p* < 0.05 was considered statistically significant.

## 3 Results

### 3.1 Basic characteristics of research participants

Among the 242 staff members, 106 were male (43.80%) and 136 were female (56.20%). The largest proportion fell within the 26–35 age group (38.00%). In terms of educational background, most held an associate degree (33.90%). Regarding professional background, the majority had majors classified as “other” (43.20%). The highest number of employees were from the logistical support department (33.50%). In terms of working hours, 64.00% of staff reported working fewer than 9 h per day. As for length of employment, the majority had 2–5 years of experience (35.10%). Details are presented in Table 1.

**Table 1:** Basic information on staff members.

Variable	Category	Frequency	Percentage (%)
Gender	Male	106	43.80
	Female	136	56.20
Age	25 years old and below	63	26.03
	26–35 years old	92	38.02
	36–45 years old	56	23.14
	46 years and above	31	12.81
Education level	High school and below	64	26.40
	Technical secondary school	30	12.40
	Associate degree	82	33.90
	Bachelor’s degree and above	66	27.30
Major	Rehabilitation Medicine	9	3.70
	Food Hygiene and Nutrition	5	2.10
	Traditional Chinese Medicine	5	2.10
	Landscape Architecture, Forestry, Agronomy	11	4.50
	Other Majors	106	43.20
Job role	Health Care Technical Services	55	22.70
	Administrative Management	50	20.70
	Equipment and Facility Maintenance	16	6.60
	Logistical Support	81	33.50
	Accounting and Finance	13	5.40
	Landscaping and Environmental Maintenance	12	5.00
	Other	15	6.20
Working hours	Less than 9 h	155	64.00
	9–12 h	72	29.80
	12 h and above	15	6.20
Years of experience	Less than 2 years	83	34.30
	2–5 years	85	35.10
	5–10 years	53	21.90
	10 years and above	21	8.70

Among the 420 consumers, 208 were male (49.50%) and 212 were female (50.50%). The largest age group was 25–35 years (31.20%). In terms of educational background, the majority held a bachelor’s degree (39.80%). Students constituted the largest occupational group among visitors (22.90%). Visitors with a monthly income below 3,000 RMB accounted for the highest proportion (32.60%). Most consumers were from within the city or nearby areas, making up 46.70% of the total. Details are presented in Table 2.

**Table 2:** Basic information on consumers.

Variable	Category	Frequency	Percentage (%)
Gender	Male	208	49.50
	Female	212	50.50
Age	18 years and below	37	8.80
	19–25 years	98	23.30
	26–35 years	131	31.20
	36–45 years	59	14.00
	46–55 years	56	13.30
	56 years and above	39	9.30
Monthly income (RMB)	Below 3,000	137	32.60
	3,000-5,000	115	27.40
	5,000-8,000	81	19.30
	8,000-15,000	58	13.80
	15,000 and above	29	6.90
Education level	Primary school and below	17	4.00
	Junior high school	56	13.30
	High school / Technical secondary school	82	19.50
	Associate degree	80	19.00
	Bachelor’s degree	167	39.80
	Master’s degree and above	18	4.30
Occupation	Government or public institution employee	74	17.60
	Enterprise employee	81	19.30
	Self-employed	55	13.10
	Worker/Agricultural laborer	17	4.00
	Freelancer	68	16.20
	Retired	18	4.30
	Student	96	22.90
	Other	11	2.60
Region of origin	Within the city and surrounding areas	196	46.70
	Within the province	147	35.00
	Other provinces and cities in China	77	18.30

### 3.2 Factors affecting the development of FHW services

#### 3.2.1 Differences in staff evaluation of the implementation of FHW services

The results (Table 3) indicated that 19 factors significantly influenced the successful implementation of FHW services (*p* < 0.05). These included understanding of the concept of FHW, awareness of relevant policies and regulations, operational models of FHW industry, perceived meaningfulness of work, participation in job training, among others.

**Table 3:** Single factor analysis of the development of FHW services.

Factor	Option	Can FHW services in Guizhou Province be effectively implemented? (*n*/%)		χ^2^	*p*
		Yes	No		
Understanding of the concept of FHW	Unfamiliar	22/59.50	15/40.50	14.897	<0.001
	Moderate	72/66.70	36/33.30		
	Familiar	84/86.60	13/13.40		
Awareness of relevant policies and regulations	Unfamiliar	36/52.20	33/47.80	31.180	<0.001
	Moderate	83/74.80	28/25.20		
	Familiar	59/95.20	3/4.80		
Operational model of FHW industry	Unfamiliar	25/54.30	21/45.70	12.217	0.002
	Moderate	65/73.90	23/26.10		
	Familiar	88/81.50	20/18.50		
Attitude of residents toward the development of FHW industry	Not supportive	1/50.00	1/50.00	34.139	<0.001
	Neutral	31/47.00	35/53.00		
	Supportive	146/83.90	28/16.10		
Effectiveness of health management programs	Poor	4/33.30	8/66.70	60.196	<0.001
	Moderate	36/46.80	41/53.20		
	Ideal	138/90.20	15/9.80		
Perceived meaningfulness of work	Disagree	4/66.70	2/33.30	10.242	0.006
	Neutral	39/59.10	27/40.90		
	Agree	135/79.40	35/20.60		
Satisfaction with professional competence	Dissatisfied	4/33.30	8/66.70	12.614	0.002
	Neutral	36/67.90	17/32.10		
	Satisfied	138/78.00	39/22.00		
Performance evaluation implemented	Yes	127/85.20	22/14.80	27.197	<0.001
	No	51/54.80	42/45.20		
Participation in work training	Yes	134/84.80	24/15.20	29.650	<0.001
	No	44/52.40	40/47.60		
Satisfaction with welfare policies	Dissatisfied	24/60.00	16/40.00	17.377	<0.001
	Neutral	50/62.50	30/37.50		
	Satisfied	104/85.20	18/14.80		
Satisfaction with FHW services	Dissatisfied	4/44.40	5/55.60	17.403	<0.001
	Neutral	32/56.10	25/43.90		
	Satisfied	142/80.70	34/19.30		
Employees’ professional competence and technical skills meet consumer needs	Yes	150/79.80	38/20.20	16.830	<0.001
	No	28/51.90	26/48.10		
Availability of traditional Chinese medicine services	Yes	126/82.40	27/17.60	16.559	<0.001
	No	52/58.40	37/41.60		
Basic infrastructure completed	Yes	165/76.00	52/24.00	6.658	0.01
	No	13/52.00	12/48.00		
Health care facilities completed	Yes	156/84.30	29/15.70	46.841	<0.001
	No	22/38.60	35/61.40		
Facility maintenance	Yes	157/77.30	46/22.70	9.283	0.002
	No	21/53.80	18/46.20		
Health education activities conducted	Yes	141/87.00	21/13.00	45.800	<0.001
	No	37/46.30	43/53.80		
Availability of health care beds	Yes	148/80.40	36/19.60	18.687	<0.001
	No	30/51.70	28/48.30		
Health record management established	Yes	124/90.50	13/9.50	46.675	<0.001
	No	54/51.40	51/48.60		

#### 3.2.2 Binary logistic regression analysis of FHW service delivery

1. Variable selection and assignment.

The staff members’ evaluation of whether FHW services in Guizhou Province can be effectively implemented was used as the dependent variable (Y). The independent variables (X) were those identified as statistically significant in the univariate analysis (see Table 3). The coding scheme for all variables is presented in Table 4.

2. Binary logistic regression results.

**Table 4:** Variable coding scheme for binary logistic regression analysis.

Variable description	Variable code	Coding scheme
Understanding of FHW concept and functions	X1	Unfamiliar = 0; Moderate = 1; Familiar = 2
Awareness of policies and regulations related to FHW	X2	Unfamiliar = 0; Moderate = 1; Familiar = 2
Knowledge of FHW industry operational models	X3	Unfamiliar = 0; Moderate = 1; Familiar = 2
Perceived support from local residents toward FHW industry	X4	Not supportive = 0; Neutral = 1; Supportive = 2
Effectiveness of health management program implementation	X5	Poor = 0; Moderate = 1; Ideal = 2
Perceived meaningfulness of work	X6	Disagree = 0; Neutral = 1; Agree = 2
Satisfaction with own professional competence	X7	Dissatisfied = 0; Neutral = 1; Satisfied = 2
Implementation of performance evaluation	X8	No = 0; Yes = 1
Participation in job training	X9	No = 0; Yes = 1
Satisfaction with FHW services	X10	Dissatisfied = 0; Neutral = 1; Satisfied = 2
Satisfaction with welfare policies	X11	Dissatisfied = 0; Neutral = 1; Satisfied = 2
Competence and technical skills to meet consumer needs	X12	No = 0; Yes = 1
Availability of Traditional Chinese Medicine services	X13	No = 0; Yes = 1
Completeness of basic infrastructure	X14	No = 0; Yes = 1
Completeness of health care facilities	X15	No = 0; Yes = 1
Maintenance of facilities	X16	No = 0; Yes = 1
Organization of health education activities	X17	No = 0; Yes = 1
Availability of health care beds	X18	No = 0; Yes = 1
Establishment of health record management	X19	No = 0; Yes = 1
Effectiveness of FHW service implementation	Y	No = 0; Yes = 1

The results (Table 5) revealed that the following factors had statistically significant impacts on staff evaluations:

1. Effectiveness of health management program implementation: OR = 4.831, 95% CI = 1.077 ~ 21.664.
2. Completeness of health care facilities: OR = 2.536, 95% CI = 1.114 ~ 5.770.
3. Organization of health education activities: OR = 3.559, 95% CI = 1.697 ~ 7.461.

**Table 5:** Binary logistic regression analysis of factors influencing the development of FHW services.

Variable	*B*	Std. error	Wald	*P*	OR	95% CI	
						Lower	Upper
Effectiveness of health management programs *(ref: Poor)*							
Moderate	−0.126	0.717	0.031	0.861	0.882	0.216	3.593
Ideal	1.575	0.766	4.232	0.040	4.831	1.077	21.664
Completeness of Health Care Facilities *(ref: No)*							
Yes	0.930	0.420	4.918	0.027	2.536	1.114	5.770
Health Education Activities Implemented *(ref: No)*							
Yes	1.269	0.378	11.296	0.001	3.559	1.697	7.461

These findings suggest that the effectiveness of health management programs, the adequacy of health care facilities, and the implementation of health education activities are key factors influencing the successful development of forest health care services in Guizhou Province.

### 3.3 Factors influencing overall satisfaction with FHW services

#### 3.3.1 Differences in consumer ratings of overall satisfaction with FHW services

The results (Table 6) indicated that 20 factors had a statistically significant impact on overall consumer satisfaction (*p* < 0.05). These factors included educational level, natural species resources, unique cultural resources, comfort of the ecological environment, et al.

**Table 6:** Univariate analysis of factors influencing overall consumer satisfaction with FHW services.

Factor	Option	Overall consumer satisfaction (*n*/%)			χ^2^	*p*
		Dissatisfied	Neutral	Satisfied		
Education level	Primary or below	1/5.9	7/41.2	9/52.9	20.791	0.023
	Junior high school	8/14.3	26/46.4	22/39.3		
	High school/Technical secondary school	15/18.3	27/32.9	40/48.8		
	Associate degree	2/2.5	26/32.5	52/65.0		
	Bachelor’s degree	22/13.2	75/44.9	70/41.9		
	Master’s degree	3/16.7	7/38.9	8/44.4		
Natural species resources	Dissatisfied	22/51.2	15/34.9	6/14.0	178.901	<0.001
	Neutral	21/13.7	102/66.7	30/19.6		
	Satisfied	8/3.6	51/22.8	165/73.7		
Unique cultural resources	Dissatisfied	21/65.6	10/31.3	1/3.1	219.819	<0.001
	Neutral	21/12.4	115/67.6	34/20.0		
	Satisfied	9/4.1	43/19.7	166/76.1		
Comfort of ecological environment	Dissatisfied	16/61.5	9/34.6	1/3.8	166.87	<0.001
	Neutral	21/14.3	99/67.3	27/18.4		
	Satisfied	14/5.7	60/24.3	173/70.0		
Promotional materials and guide maps	Dissatisfied	21/70.0	8/26.7	1/3.3	217.407	<0.001
	Neutral	21/12.4	113/66.9	35/20.7		
	Satisfied	9/4.1	47/21.3	165/74.7		
Designated wellness zones	Dissatisfied	22/56.4	14/35.9	3/7.7	215.208	<0.001
	Neutral	17/10.4	115/70.6	31/19.0		
	Satisfied	12/5.5	39/17.9	167/76.6		
Health care service facilities	Dissatisfied	23/59.0	13/33.3	3/7.7	241.252	<0.001
	Neutral	24/14.9	112/69.6	25/15.5		
	Satisfied	4/1.8	43/19.5	173/78.6		
Main road layout	Dissatisfied	23/65.7	8/22.9	4/11.4	197.022	<0.001
	Neutral	16/10.9	101/68.7	30/20.4		
	Satisfied	12/5.0	59/24.8	167/70.2		
Layout of leisure facilities (e.g., Pavilions)	Dissatisfied	24/70.6	9/26.5	1/2.9	249.543	<0.001
	Neutral	18/12.8	103/73.0	20/14.2		
	Satisfied	9/3.7	56/22.9	180/73.5		
Accessibility of scenic areas	Dissatisfied	26/72.2	7/19.4	3/8.3	256.101	<0.001
	Neutral	15/10.6	105/73.9	22/15.5		
	Satisfied	10/4.1	56/23.1	176/72.7		
Professionalism of wellness instructors/therapists	Dissatisfied	30/57.7	19/36.5	3/5.8	249.878	<0.001
	Neutral	18/11.3	109/68.1	33/20.6		
	Satisfied	3/1.4	40/19.2	165/79.3		
Courtesy of service personnel	Dissatisfied	32/80.0	6/15.0	2/5.0	296.768	<0.001
	Neutral	15/9.5	109/69.0	34/21.5		
	Satisfied	4/1.8	53/23.9	165/74.3		
Professional knowledge of service staff	Dissatisfied	33/71.7	10/21.7	3/6.5	304.792	<0.001
	Neutral	14/9.2	111/73.0	27/17.8		
	Satisfied	4/1.8	47/21.2	171/77.0		
Overall competence of service staff	Dissatisfied	24/77.4	6/19.4	1/3.2	270.197	<0.001
	Neutral	16/10.5	113/73.9	24/15.7		
	Satisfied	11/4.7	49/20.8	176/74.6		
Diversity of health care programs	Dissatisfied	26/61.9	12/28.6	4/9.5	231.796	<0.001
	Neutral	19/11.7	111/68.5	32/19.8		
	Satisfied	6/2.8	45/20.8	165/76.4		
Experiential quality of health care programs	Dissatisfied	25/73.5	6/17.6	3/8.8	284.92	<0.001
	Neutral	20/12.1	120/72.7	25/15.2		
	Satisfied	6/2.7	42/19.0	173/78.3		
Perceived benefits after forest health care	Dissatisfied	26/72.2	10/27.8	0/0.0	267.959	<0.001
	Neutral	17/10.2	117/70.1	33/19.8		
	Satisfied	8/3.7	41/18.9	168/77.4		
Special forest health care projects	Dissatisfied	18/64.3	9/32.1	1/3.6	185.437	<0.001
	Neutral	22/13.9	105/66.5	31/19.6		
	Satisfied	11/4.7	54/23.1	169/72.2		
Special forest health care accommodation	Dissatisfied	16/61.5	7/26.9	3/11.5	199.894	<0.001
	Neutral	28/15.4	120/65.9	34/18.7		
	Satisfied	7/3.3	41/19.3	164/77.4		
Availability of health product purchase channels	Dissatisfied	31/68.9	10/22.2	4/8.9	284.59	<0.001
	Neutral	17/9.3	124/68.1	41/22.5		
	Satisfied	3/1.6	34/17.6	156/80.8		

#### 3.3.2 Ordered logistic regression analysis of factors influencing overall satisfaction

1. Variable selection and assignment

Overall consumer satisfaction with FHW services was used as the dependent variable (Y). Independent variables (X) included those found to be statistically significant in the univariate analysis (see Table 6). The coding scheme for all variables is detailed in Table 7.

2. Ordered logistic regression results

**Table 7:** Coding scheme for ordered logistic regression analysis.

Variable dimension and name	Variable code	Coding scheme
Education level	X1	Primary school or below = 0; Junior high = 1;
		High school/Technical secondary = 2; Associate degree = 3;
		Bachelor’s degree = 4; Master’s degree or above = 5
Natural species resources	X2	Dissatisfied = 0; Neutral = 1; Satisfied = 2
Unique cultural resources	X3	Dissatisfied = 0; Neutral = 1; Satisfied = 2
Comfort of ecological environment	X4	Dissatisfied = 0; Neutral = 1; Satisfied = 2
Promotional materials and guide maps	X5	Dissatisfied = 0; Neutral = 1; Satisfied = 2
Designated wellness zones	X6	Dissatisfied = 0; Neutral = 1; Satisfied = 2
Health care service facilities	X7	Dissatisfied = 0; Neutral = 1; Satisfied = 2
Rationality of main road layout	X8	Dissatisfied = 0; Neutral = 1; Satisfied = 2
Layout of leisure facilities (e.g., Pavilions)	X9	Dissatisfied = 0; Neutral = 1; Satisfied = 2
Accessibility of scenic areas	X10	Dissatisfied = 0; Neutral = 1; Satisfied = 2
Professionalism of wellness instructors/therapists	X11	Dissatisfied = 0; Neutral = 1; Satisfied = 2
Courtesy of service staff	X12	Dissatisfied = 0; Neutral = 1; Satisfied = 2
Professional knowledge of service staff	X13	Dissatisfied = 0; Neutral = 1; Satisfied = 2
Overall competence of service staff	X14	Dissatisfied = 0; Neutral = 1; Satisfied = 2
Diversity of health care programs	X15	Dissatisfied = 0; Neutral = 1; Satisfied = 2
Experience quality of health care programs	X16	Dissatisfied = 0; Neutral = 1; Satisfied = 2
Perceived benefits after FHW experience	X17	Dissatisfied = 0; Neutral = 1; Satisfied = 2
Special FHW projects	X18	Dissatisfied = 0; Neutral = 1; Satisfied = 2
Special FHW accommodations	X19	Dissatisfied = 0; Neutral = 1; Satisfied = 2
Availability of health product purchase channels	X20	Dissatisfied = 0; Neutral = 1; Satisfied = 2
Overall consumer satisfaction	Y	Dissatisfied = 0; Neutral = 1; Satisfied = 2

The ordered logistic regression results (Table 8) indicate that the professionalism of wellness guides/therapists, consumers’ perceived gains following the forest-wellness experience, the rational placement of amenities (e.g., rest pavilions), courteous staff service, and the availability of health-product purchasing channels are significant determinants of overall satisfaction with FHW services.

**Table 8:** Ordered logistic regression analysis of factors influencing overall consumer satisfaction.

Variable	*B*	Std. error	Wald	*p*	OR	95% CI	
						Lower	Upper
Layout of leisure facilities (ref: satisfied)							
Dissatisfied	−1.009	0.792	1.624	0.202	0.365	0.077	1.717
Neutral	−0.802	0.384	4.367	0.037	0.449	0.211	0.952
Professionalism of instructors/therapists (ref: satisfied)							
Dissatisfied	−2.339	0.650	12.958	<0.001	0.096	0.027	0.347
Neutral	−1.068	0.400	7.144	0.008	0.344	0.157	0.753
Courtesy of service personnel (ref: satisfied)							
Dissatisfied	−1.853	0.792	5.472	0.019	0.157	0.033	0.745
Neutral	0.177	0.401	0.196	0.658	1.194	0.544	2.623
Perceived benefit after experience (ref: satisfied)							
Dissatisfied	−1.564	0.778	4.041	0.044	0.209	0.046	0.958
Neutral	−0.161	0.432	0.140	0.709	0.851	0.364	1.989
Availability of product purchase channels (ref: satisfied)							
Dissatisfied	−3.163	0.682	21.518	<0.001	0.042	0.011	0.164
Neutral	−1.017	0.385	6.960	0.008	0.362	0.170	0.770

## 4 Discussions and recommendations

### 4.1 Adhering to the concept of increasing efficiency in health management, optimizing the design of FHW services

Health management is not only a form of disease control but also a health promotion initiative. It emphasizes that the most effective methods should be applied to the design, implementation, and evaluation of each component of the program (37). Evidence suggests that health management can improve health outcomes and yield economic benefits, for instance, every one-dollar investment in health management may save up to 3.24 dollars in related costs (38). An ideal health management program tailors personalized health plans for different clients, effectively improving their health conditions—such as regulating bodily functions and alleviating chronic disease symptoms—thus enhancing their experience and health benefits during FHW activities. This study found that the more closely the implementation of health management programs aligned with this ideal state, the more effectively FHW services were delivered in Guizhou Province. Related studies have also shown that health management can significantly improve the health status and disease management capabilities of older population individuals with hypertension and diabetes (39). Therefore, integrating ideal health management programs into FHW services can enhance client experience and health benefits, thereby improving the overall level of service delivery in the market.

To better advance FHW services, FHW bases and relevant health organizations should establish a health management closed loop centered on “comprehensive assessment–personalized intervention–continuous follow-up,” thereby creating a generalizable and scalable practice framework that enables a holistic understanding of clients’ health status and targeted, evidence-based interventions, ensuring the scientific validity and effectiveness of health management plans. In parallel, providers should leverage “Internet+” and digital health technologies to innovate service models encompassing online health assessments, remote health coaching, health-data management platforms, and smart health devices, thereby improving convenience and accessibility, expanding service coverage, and enhancing operational efficiency. Moreover, cross-regional collaboration with research institutes and universities should be strengthened to advance health-management research and translate findings into practice, providing a robust scientific foundation for the institutionalization and sustainable development of FHW services.

This study also found that consumer satisfaction with the perceived “benefits gained” after participating in FHW programs had a significant positive impact on their overall satisfaction. Such perceived gains are not limited to temporary relaxation but extend to tangible improvements in physical and mental well-being. Related studies have also shown that FHW experiences can effectively relieve stress and improve emotional states (40), which undoubtedly contribute to higher long-term consumer satisfaction. To better meet consumers’ personalized demand for tangible gains, several countries have begun exploring product customization. For example, Germany provides diversified FHW offerings and bespoke therapeutic/convalescence packages tailored to different population groups, ensuring that each visitor receives the most suitable wellness experience (41). This implies that forest-wellness providers should emphasize program effectiveness and specificity in project design so that participants obtain concrete health benefits and psychological well-being.

Accordingly, we recommend that FHW bases and relevant health organizations innovate program design concepts, align offerings precisely with user needs, and establish a highly adaptive program-development system capable of meeting diverse demands across different market contexts. Specifically, providers should conduct in-depth assessments of the health needs and preferences of distinct consumer segments and design more targeted programs to ensure that participants achieve individualized health improvements and psychological well-being. By offering a diversified portfolio of options, providers can better match heterogeneous consumers, thereby enhancing both program effectiveness and generalizability. In parallel, institutions should institute routine feedback and evaluation mechanisms to dynamically adjust program content based on service outcomes and user feedback, enabling continuous optimization of program design and improving participation and appeal.

### 4.2 Enhance the service level of wellness talents and optimize the training mode of professional talents

The study found that the professionalism of FHW instructors or guides had a significant impact on overall consumer satisfaction. This suggests that professional explanations and guidance can effectively enhance service satisfaction. Furthermore, courteous and attentive service was also shown to positively influence consumer satisfaction—a finding consistent with the research results of scholar Xiong Linman (42). These two aspects underscore the critical role that service quality plays in shaping a positive consumer experience. International models of FHW likewise underscore the importance of service quality. For example, the Association of Nature and Forest Therapy (ANFT)—a global leader in guide training—places strong emphasis on building professional capacity within service teams and has trained approximately 2,400 certified guides to date (43). These professionally trained guides provide precise, evidence-informed interpretation and facilitation, thereby ensuring the professionalism and standardization of forest-wellness activities and enabling more people to experience high-quality FHW services.

To enhance the quality of FHW services, FHW bases and relevant health organizations should prioritize upgrading workforce competence and service standards, and establish a transferable framework for talent cultivation and quality assurance. Firstly, implement systematic training for relevant personnel, with curricula covering foundational FHW knowledge, health-management competencies, emergency response, and humanistic/compassionate communication, delivered via pre-service training, in-service training, and professional certification to ensure teams can provide professional, high-quality services. Secondly, promote university–industry collaboration and integration of education with practice: enterprises and universities should co-establish talent-training bases (enterprises offering practice platforms and industry mentors; universities providing theoretical instruction and technical support), jointly design curricula and training schemes that embed sector needs into teaching, and Cultivating professionals with multidisciplinary capabilities. In parallel, education authorities should guide universities in tourism management, health services, and related disciplines to innovate application-oriented training models that tightly integrate theory and practice, thereby meeting the sector’s demand for innovative talent (44). Thirdly, optimize service processes and touchpoints by instituting standardized service protocols, emphasizing courteous language, professional appearance, and communication skills, building a high-quality service brand and enhancing consumer experience; complement these measures with robust performance appraisal systems and diversified incentives to drive continuous improvement. Finally, establish enduring supervision and feedback mechanisms to ensure compliance with quality standards and continuous quality enhancement.

### 4.3 Expanding health product/service channels to create a convenient and secure consumption experience

In FHW services, health products refer broadly to goods and services that promote physical and mental well-being (19), encompassing tangible goods such as health foods, medical devices, and fitness equipment, as well as service-oriented products like specialized diagnostic and therapeutic techniques, physical exercise programs, and cultural or tourism-related services. The findings of this study indicate a positive correlation between the availability of health product purchase channels and overall consumer satisfaction. This suggests that expanding access to health products is essential for creating a convenient and secure consumer experience, thereby enhancing satisfaction.

To achieve this goal, we recommend that FHW bases and related health organizations integrate online and offline resources to build a multi-channel sales and service network, while simultaneously strengthening quality management and brand development. Specifically, providers should establish on-site health-product experience stores within bases or wellness centers that combine display, consultation, and sales to enhance consumers’ direct engagement; in parallel, they should open official flagship or specialty stores on major e-commerce platforms to leverage large user traffic for broader reach, and make full use of new-media and social-commerce platforms (e.g., live streaming and short-video marketing) to meet anytime, anywhere purchasing needs. At the same time, a standardized quality-management system should be instituted to exercise end-to-end control over production, processing, and distribution, ensuring product safety and efficacy; a product traceability mechanism and robust after-sales service system should be established to bolster consumer trust and protect consumer rights. In addition, sustained, multi-channel outreach and interactive promotion can strengthen brand building; active participation in health expos and regional product showcases can highlight product features and advantages, increase visibility and influence; and routine consumer-engagement activities can deepen emotional connection and foster customer loyalty, thereby cultivating a strong reputation.

### 4.4 Accelerate the improvement of health care facilities and optimize the infrastructure layout of the base

Facility construction is a critical component of both the development and certification of FHW bases. These facilities primarily serve to support a range of health care services, while also fulfilling functions related to leisure and recreation, nature immersion, science education, specialized physical activities, and patriotic education (45) The study revealed that the completeness of health care facilities significantly affects the implementation of FHW services—a finding consistent with the results of Song Mengke (46). Furthermore, the reasonable layout of facilities such as pavilions plays an important role in enhancing overall consumer satisfaction, aligning with the research of Wu Baijun et al. (47). These findings demonstrate that well-designed and logically arranged facilities can improve consumers’ overall evaluations of FHW services. International experience also underscores the importance of facility design and layout for enhancing user experience. For example, in Japan, forest-therapy programs prioritize safety, comfort, and psychotherapeutic effects: trail gradients are strictly controlled, appropriate widths are maintained, shrubbery and vines along the path are cleared, and routes include viewing/rest areas and interpretive signage (48). These practices explicitly address users’ physiological and psychological needs, creating a safe, comfortable, and restorative forest environment. Accordingly, when upgrading health care facilities, providers should focus not only on the number and basic functions of installations but also on design details and user experience, thereby improving overall service quality and, in turn, consumers’ assessments of the service.

To maximize the effectiveness of health care facilities, FHW bases and related institutions should adopt master planning with phased implementation. Planning should be grounded in rigorous assessments of market demand and local resource endowments, prioritize the construction of core wellness functional zones, and be supported by scientifically sound timelines and budget plans to ensure controllable and sustainable project delivery. Equal emphasis should be placed on the rational layout of leisure amenities—harmonized with topography and landscape—to create comfortable rest environments and maintain coherence with the surrounding natural setting. During facility development, the needs of diverse user groups, including those with special requirements, should be fully considered to ensure an accessible, convenient wellness experience for all. Finally, routine inspection and maintenance should be institutionalized, with timely repair of defects to guarantee the safe and stable operation of facilities to provide visitors with a reliable environment for FHW.

### 4.5 Using health education activities as a tool to help improve FHW services

Whitehead defines health education as an activity aimed at informing individuals about the nature and causes of health and illness, as well as the personal risks associated with behaviors linked to lifestyle choices (49). Health education seeks to directly influence individuals’ value systems, beliefs, and attitudes in order to motivate behavioral change—particularly when individuals face high health risks or are already affected by disease or disability. Studies have shown that health education interventions can significantly improve diabetes patients’ understanding of disease-related knowledge, foster healthier lifestyle habits, and help maintain blood glucose levels within a normal range (50, 51). These findings offer valuable insights for integrating health education into FHW services. This study found that the implementation of health education activities is an important driver in advancing FHW services.

Building on these findings, firstly, we recommend that FHW bases and related institutions continuously innovate the formats of health-education activities by integrating experiential programming, interactive games, online courses, and digital/smart-health technologies to enhance attractiveness and engagement. Secondly, tailor stratified, population-specific themes according to health status and age to enable individualized, targeted delivery; and strengthen interdisciplinary collaboration by engaging medical experts, nutritionists, psychologists, and rehabilitation specialists to provide authoritative lectures, counseling, and guidance. In terms of dissemination, a multi-channel, routine communication mechanism should be established—leveraging new media, traditional media, and community platforms—to sustain the promotion of forest-wellness concepts and health knowledge and expand societal reach. Finally, health-education activities should be institutionalized on a regular schedule to ensure continuous access to information and services, thereby improving public understanding of FHW and stimulating service demand.

This study’s findings carry both academic value and real-world implications. First, the results provide reference for governments and policymakers, helping them pay greater attention to regional characteristics and local advantages when promoting the development of the FHW industry, thereby formulating more targeted policies to ensure sustainability. Second, the identified determinants have direct relevance for managers and service providers of FHW bases, enabling them to optimize program content and improve resource allocation efficiency to enhance service quality and customer satisfaction. Third, the findings inform efforts to promote synergies between the health industry and ecological conservation, supporting win–win outcomes for economic and ecological benefits. Finally, this study provides an empirical foundation for subsequent scholars, laying the groundwork for comparative studies across broader contexts or different regions, thereby further extending the depth and breadth of FHW research.

## 5 Threats to validity

Although this study was designed with methodological rigor, several validity threats should be acknowledged. Internal validity may be limited by the cross-sectional survey design, which captures attitudes and behaviors at a single point in time and restricts causal inference. External validity is constrained by the sampling frame, as data were collected primarily from FHW bases in Guizhou Province; while representative of the local context, the findings may not be fully generalizable to other regions or countries. Construct validity may be affected by the reliance on self-reported measures, which are subject to potential response bias and may not fully capture underlying constructs. Conclusion validity could be influenced by the exclusive use of quantitative methods, which limits the depth of interpretation regarding individual experiences and motivations.

Recognizing these threats does not undermine the contribution of this study; rather, it underscores the need for future research to adopt longitudinal designs, expand sampling across diverse contexts, and incorporate mixed-methods approaches to strengthen the robustness and applicability of findings.

## 6 Conclusion

By analyzing FHW bases in Guizhou Province from both the supply and demand perspectives, this study identifies the key determinants of FHW service development. On the supply side, the effectiveness of health-management programs, the completeness and layout of wellness facilities, and the regular implementation of health-education activities significantly shape service delivery and quality. On the demand side, overall consumer satisfaction is driven by the professionalism of FHW instructors or therapists, perceived gains from participation, the rational siting of leisure amenities, courteous staff service, and the availability of channels for purchasing health-related products. Together, these findings indicate that comprehensive health management, professional service teams, user-centered facility environments, and convenient product access are central to enhancing the consumer experience and improving service performance. This study contributes to the literature by jointly examining supply- and demand-side determinants within a single empirical framework, thereby providing a more comprehensive understanding of the mechanisms underpinning FHW service development and generating policy-relevant, practice-oriented insights.

Building on these results, we recommend that FHW bases optimize service design, enhance service quality, expand product supply channels, improve facility development and layout, and institutionalize health-education activities to promote the sustainable development of FHW services. These recommendations can inform scalable, evidence-informed practices and policy actions in China and comparable international contexts.

Despite these contributions, several limitations should be acknowledged. First, the cross-sectional survey design captures attitudes and behaviors at a single point in time, limiting causal inference and the characterization of dynamics among variables. Second, the sample is drawn primarily from FHW bases in Guizhou; while reasonably representative, the findings may not fully generalize to other regions or countries. Third, the analysis relies mainly on quantitative methods and lacks qualitative exploration of individual experiences and underlying motivations, which may constrain the depth and comprehensiveness of the conclusions. Future research should incorporate longitudinal data, broaden the sampling frame, and employ mixed-methods designs that integrate quantitative and qualitative approaches to enhance the robustness and generalizability of the evidence.

## Data Availability

The original contributions presented in the study are included in the article/supplementary material, further inquiries can be directed to the corresponding author.
